# Susceptibility of primary, secondary and suspected vectors to *Plasmodium vivax* and *Plasmodium falciparum* infection in Ethiopia

**DOI:** 10.1186/s13071-022-05467-5

**Published:** 2022-10-21

**Authors:** Arega Tsegaye, Assalif Demissew, Dawit Hawaria, Hallelujah Getachew, Kassahun Habtamu, Abebe Asale, Guiyun Yan, Delenasaw Yewhalaw

**Affiliations:** 1 Department of Biology, College of Natural Science, Jimma University, Jimma, Ethiopia; 2 Department of Medical Laboratory Sciences, College of Medicine and Health Sciences, Ambo University, Ambo, Ethiopia; 3 School of Medical Laboratory Sciences, Faculty of Health Sciences, Jimma University, Jimma, Ethiopia; 4 Department of Medical Laboratory Sciences, Arbaminch College of Health Sciences, Arbaminch, Ethiopia; 5 School of Public Health, Hawassa University, Hawassa, Ethiopia; 6 Department of Medical Laboratory Sciences, Menelik II College of Medicine and Health Science, Kotebe University of Education, Addis Ababa, Ethiopia; 7 International Centre of Insect Physiology and Ecology (ICEPE), LRI Campus, Gurd Shola, Addis Ababa, Ethiopia; 8 Program in Public Health, University of California at Irvine, Irvine, CA 92697 USA; 9 Tropical and Infectious Diseases Research Center (TIDRC), Jimma University, Jimma, Ethiopia; 10 Department of Microbial, Cellular & Molecular Biology, Addis Ababa University, Addis Ababa, Ethiopia

**Keywords:** *Plasmodium* species, *Anopheles*, Malaria, Membrane feeding assay, Infection rate, Oocysts, Ethiopia

## Abstract

**Background:**

Insecticide-based vector control interventions in combination with case management with artemisinin-based combination therapy has reduced malaria incidence and prevalence worldwide. Current control methods focus on the primary malaria vectors, *Anopheles gambiae* sensu lato (s.l.) and the *An. funestus* group; however, the impact of secondary and suspected vectors has been either sidelined or received limited attention. Defining the susceptibility of secondary, suspected vector species to different parasites in time and space is essential for efficient malaria control and elimination programs. The aim of this study was to assess the susceptibility of *An. gambiae* s.l., *An*. *coustani* complex and *An*. *pharoensis* to *Plasmodium vivax* and *P. falciparum* infection in Ethiopia.

**Methods:**

Larvae of *Anopheles* spp. were collected from different aquatic habitats and reared to adults under laboratory conditions, with the temperature and humidity maintained at 27 ± 1 °C and 75 ± 5%, respectively. Adult female mosquitoes were identified to species as *An. gambiae* s.l., *An. coustani* complex and *An*. *pharoensis*. Females of these three *Anopheles* spp. were allowed to feed in parallel feeding assays on infected blood containing the same gametocytes isolated from* P. falciparum* and* P. vivax* gametocyte-positive patients by indirect membrane feeding assays. All blood-fed mosquitoes were held under laboratory conditions. After 7 days, all surviving mosquitoes were dissected to detect mid-gut oocyst and enumerated under a microscope.

**Results:**

Of 5915 female *Anopheles* mosquitoes exposed to gametocyte-infected blood, 2106 (35.6%)s fed successfully in the 32 independent infection experiments. There was a significant variation in feeding rates among *An. gambiae* s.l., *An. pharoensis* and *An. coustani* complex (G-test = 48.43, *P* = 3.049e-11). All three exposed mosquito species were receptive to *P. vivax* and *P. falciparum* infection development. The percentage of infected mosquitoes following feeding on an infected blood meal was significantly different among species (G-test = 6.49, *P* = 0.03886). The median infection intensity (II) for *An. coustani* complex, *An. gambiae* s.l. and *An. pharoensis* was 1.16, 2.00 and 1.25, respectively. Although the proportion of infected mosquitoes significantly differed in terms of II, infection rate (IR) and mean oocyst density among the species, mean oocyst density and IR were highly correlated with gametocyte density in all tests (*P* < 0.001).

**Conclusion:**

Primary, secondary and suspected vectors were experimentally susceptible to both *P. vivax* and *P. falciparum* infection. An effective malaria elimination program might include surveillance and control tools which target secondary and suspected vectors that might play an outdoor transmission role, possibly resulting in reduced focal malaria transmission.

**Graphical Abstract:**

**Figure:**
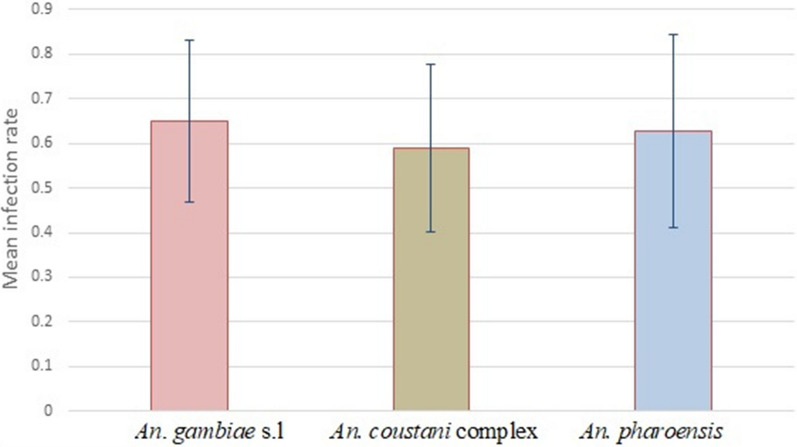
Comparison of the three species’ mean infection rates with standard deviation

## Background

Malaria continues to be a major public health challenge worldwide, with 241 million estimated cases and 627,000 deaths in 2020, of which 96% were reported from the WHO African region [1]. In Ethiopia, despite a considerable decline of malaria morbidity in recent years [1–3], the disease remains a focus of public health concern, with 1.8 million malaria cases reported in 2020 [1]. In sub-Saharan Africa, *Plasmodium vivax* and *Plasmodium falciparum* almost consistently co-occur and—in the epidemiological context—are equally important, although the latter is the most virulent of the two species [4]. In the last decades, most disease cases were attributed to *P. falciparum*, which is responsible for 90% of malaria-related deaths. In recent years, however, *P. vivax* has been reported to cause malaria complications among children in endemic regions [4–6].

In Ethiopia, there are more than 47 documented species of *Anopheles* mosquitoes [7, 8] of which *Anopheles arabiensis*, *An. pharoensis, An. funestus* and *An. nili* are recognized malaria vectors. The primary malaria vector is *An. arabiensis* while *An. pharoensis*, *An. funestus* and *An. nili* are secondary vectors that occur at varying densities, with limited distribution and vector competency [9, 10]. More recently, a new invasive *Anopheles* species, *An. stephensi*, has been documented in the country [11]. Populations of *Anopheles* mosquitoes, such as *An*. *arabiensis*, *An. amharicus*, *An. pharoensis*, *An. coustani, An. nilli*,* An. funestus* group and* An. squamouse*, usually co-occur in the East Wollega zone, western Ethiopia [12–14]. However, *An*. *arabiensis*, *An. amharicus, An. coustani and An. pharoensis* populations occur abundantly both in the rainy and dry season in western parts of Ethiopia [12].

Environmental modifications and water development projects may increase the risk of malaria transmission by contributing to the formation of additional breeding habitats with changes in micro-weather conditions and micro-ecological settings [15, 16]. Malaria distribution in different ecological settings is largely governed by the spatial and temporal distribution of malaria vectors [9]. The construction of irrigation schemes creates additional aquatic habitats, modifying the ecology of the area to conditions favoring mosquito breeding [17]. Such ecological change may also lead to shifts in mosquito fauna, distribution, abundance, vector diversity and proliferation [12, 13].

During the dry season, malaria transmission in irrigated areas and areas resulting from dam construction is supported by primary vectors, even though the abundance of secondary vectors is also relatively high [12]. The contribution of secondary and suspected vectors has not been well studied. However, the abundance and distribution of secondary and suspected vectors has increased considerably over the years [18], with modifications to environments creating new larval habitats [19]. However, the impact of this changed ecological setting due to the introduction of irrigation schemes and sugar plantations on the bionomics of *Anopheles* mosquitoes and their vector competence is not well studied in these areas.

In Ethiopia, studies on the role of secondary vectors are limited or missing entirely, possibly due to the extremely low infection rate (IR) or because no infections were reported from the field-collected adult mosquitoes that made up most of the collections [20, 33]. In addition, existing vector surveillance methods mainly focus on indoor resting and indoor biting species, with prioritization of the anthropophagic behavior and vectorial capacity of the primary vector [21, 22]. Data on secondary vectors is limited, and findings from the limited number of studies currently available indicate that most secondary vectors do have a short survival rate, with a natural mortality rate likely to be around 50–60% per gonotrophic cycle [23, 24]. However, experimental vector susceptibility studies that evaluate the vector capacity of suspected mosquito species are limited in Ethiopia [32]. Studies conducted in Ethiopia and the neighboring country of Kenya on wild-caught adult mosquitoes indicate that *An. coustani* is susceptible to *Plasmodium* infection [18, 33, 37]. Defining the receptiveness of *Anopheles* spp. to *Plasmodium* spp. in time and space is important in vector control programs, but few experimental infection studies on secondary and suspected vectors over both time and space have been conducted in Ethiopia. Therefore, the aim of the study reported here was to assess the susceptibility of *Anopheles gambiae* sensu lato (s.l.), the *An. coustani* complex and *An. pharoensis* to *P. vivax and P. falciparum* infection using indirect membrane feeding assay at the Arjo-Didessa irrigation scheme, south-western Ethiopia.

## Methods

### Study area

The study was conducted on the Arjo-Didessa sugarcane plantation irrigation scheme, southwest Ethiopia (8°41′35.5″N 36°25′54.9″E), located 575 km southwest of the capital city of Ethiopia, Addis Ababa. The altitude ranges from 1300 to 2280 m a.s.l., with a mean annual rainfall of 1477 mm that is distributed over a short rainy season between February and April and a long rainy season between June and September, corresponding to the low and high peak transmission seasons, respectively. The soil type at the Arjo-Didessa sugarcane plantation site is clay and clay loam with low porousness. Due to this slow rate of percolation, rainwater can accumulate in the top layers of the soil and form swamps in the area [25], creating a wide range of breeding sites for malaria vector mosquitoes. The study site is in a malaria-endemic area, and *P. falciparum* and *P. vivax* are the principal malaria parasite species responsible for the majority of the infections. Various *Anopheles* mosquitoes, including *An. arabiensis*, *An. amharicus*, *An. pharoensis, An. coustani* complex and *An. squamous*, occur in this area [12, 14].

### Mosquito collection, rearing and identification

*Anopheles* larvae and pupae were collected from natural breeding habitats of the irrigation clusters and surrounding villages, and blood-fed adult and gravid mosquitoes for egg-laying were collected at dawn from human dwellings and animal indoor resting sites (sheds) using mouth aspirators. Field-collected adult mosquitoes were only used for oviposition purposes. Larvae and pupae were collected using a standard dipper (350 ml; BioQuip Products, Inc., Compton, CA, USA), immediately transported to the International Centre of Excellence for Malaria Research (ICEMR) field laboratory and reared to adults in enamel trays (27 × 16 × 6.5 cm). Immediately after collection, mosquito larvae were filtered remove competitors, predators and unwanted debris, transported to the insectary in water taken from the mosquito’s normal breading aquatic habitat and reared to adult stages on fish food (TetraMin; Tetra Werke, Melle, Germany). The three species under study were sorted and kept separately in different cages; all cages were provided daily with a 10% sucrose solution as a source of energy. Adult mosquitoes collected from the field were transferred for oviposition and rearing processes [26]. The eggs laid on filter papers were washed onto enamel trays and allowed to stand until emergence. Larvae were fed with fish food (TetraMin Baby; Tetra Werke). Pupae were transferred to cups containing water and allowed to develop to adults inside cages. The emerged adults were fed on cotton wool soaked in 10% sucrose solution. Rearing was done under laboratory conditions (25 ± 2 °C, 70% ± 10% relative humidity) [27].

### Patient recruitment and sample collection

Persons suspected of having malaria, were febrile and had visited health facilities were eligible for enrollment in the study. As a first step, blood was obtained by finger prick from febrile patients who were screened for malaria infection at any of five health facilities (Arjo Diddessa Sugar Factory clinic, Abote Didessa Health Post, Kerka Health Post, Command Two Health Post and Command Five Heath Post), and examined as Giemsa-stained blood smear. Among these patients who tested positive for malaria, individuals diagnosed with malaria at the stage in which the parasite was circulating in the blood at the gametocyte stage were asked to consent to be enrolled in the study. Patients who refused to consent, had severe illness and/or were mentally sick, as well as those who had taken anti-malaria drugs within 2 weeks prior to screening were excluded from the study. All patients who agreed to participate in the study were transferred to the ICEMR Laboratory where venous blood was sampled and collected into heparin tubes [28].

Samples used for the membrane feeding assay were double screened (first for *Plasmodium* infection, then for the presence of gametocytes in the blood). Parasite and gametocyte densities were the blood smears were determined per leukocyte and assuming 8000 leukocytes/μl of blood. The sexual and asexual stages of the parasite were determined per leukocyte, against 500 and 200 leukocytes/μl, respectively. The time when the patient carrying gametocytes was available was not known; however, mosquitoes were always available for the membrane feeding assays to perform experimental infection on the same day. All persons who had fever during the physical examination and who tested positive for malaria parasites on the blood film examination were treated immediately as per the national malaria treatment guidelines [29].

### Indirect membrane feeding assay

For the indirect membrane feeding assay (IDMFA), adult female mosquitoes aged 3–5 days were starved 8–12 h prior to membrane feeding as this was believed to increase the blood-feeding activity of the mosquitoes. The mosquitoes were provided with infected blood from patients who had tested positive for *P. falciparum* or *P. vivax* for a period of 30–60 min via an artificial membrane feeding apparatus; micro-glass feeders were used for experimental mosquito infection. Glass feeders were closed with membrane and placed on top of paper cups covered the meshes size (8.5/11 cm, diameter/depth) with starved mosquitoes. During the feeding process, the temperature was kept constant at 37 ± 0.1 °C using a high-speed water circulation system-connected to the glass feeders, which allowed the mosquitoes of the three species to feed in parallel and up to 450 mosquitoes to feed at one time [30].

An artificial membrane feeding apparatus with a water pump (model 8005; PolyScience, Niles, IL, USA) was connected to the nine glass feeders, and each feeder was filled with 200 μl of freshly drawn infected blood using a blunt needle. The starved mosquitoes in the paper cups enclosed within a mesh were then allowed to feed on infected blood from the same source for a period of 30–60 min under reduced light conditions. A total of nine paper cups were used in a single experiment; in each paper cup 50 starved mosquitoes, with three replicates for each species and three species, were allowed to feed in parallel on the same infected blood source [30]. Feeding success was measured after 30 min, at which time the glass feeders were detached and unfed, partially fed mosquitoes were killed and discarded. Fully fed mosquitoes were maintained at 25 ± 2 °C and 70 ± 10% relative humidity. Until day 8 post-feeding, infected mosquitoes were provided with cotton wool soaked in 10% sucrose solution.

### Mosquito dissection

Eight days after feeding on infected blood, all experimentally infected and surviving mosquitoes were immobilized and dissected, and then examined for oocyst development on the midgut and enumerated. Midguts were removed, and oocyst counting was performed by examining the wet mount midgut stained with a 5% mercurochrome solution [30, 31]. Oocysts from the midgut were examined and enumerated under a microscope with a 40× objective. For each infection assay, the infection intensity (II) and IR were determined.

### Data analysis

The R statistical software package version (4.2.0) was employed to analyze data. The *P. vivax* and *P. falciparum* IR for *An. gambiae* s.l., *An. coustani* complex and *An. pharoensis* was assessed by the presence of oocysts in the midguts. The IR was calculated as IR = $\frac{{\text{oocyst positive mosquitoes}} }{{\text{total mosquitoes dissected in individual feeding}} }  \times 100$. Similarly, the feeding rate was calculated as FR = $\frac{{\text{Fed mosquitoes}}}{{\text{total exposed}}} \times 100.$

The FR and infection frequency between *Anopheles* spp. were compared using the G-test; species pairwise comparisons were also computed. The overall II and IR were compared between populations of *An. gambiae* s.l., *An. coustani* complex and *An. pharoensis* using Pearson’s Chi-square test and the Kruskal–Wallis test, respectively. Gametocyte density with IR and mean oocyst number were correlated using Spearman’s rank correlation test.

## Results

### *Anopheles* mosquito exposure, feeding, survival and IR by the IDMFA

Overall, 586 febrile patients were screened for malaria during the study period. Of these, 94 individuals were unwilling to participate and 57 had taken anti-malarial drugs within the 48 h prior to sample collection and were excluded. Malaria screening confirmed that 125 patients had malaria, of whom 54 (43.2%) were infected with *P. vivax* and 71 (56.8%) with *P. falciparum*. Of these 54 and 71 patients, 40 (74%) and 19 (26.7%), respectively, were confirmed by microscopic examination of the blood smears to be gametocyte carriers.

Although all persons confirmed to be gametocyte carriers satisfied the inclusion criteria and were enrolled in the experimental infection study, only 32 parallel successful mosquito feeding assays were considered for analysis. Of these, 32 feeding experiments, 24 (68.6%) were infected with *P. vivax* and eight (31.4%) were infected with *P. falciparum.* The three mosquito species differed in feeding time, with *An. gambiae* s.l. feeding quickly (35% of individuals fully engorged in 30 min) and *An. pharoensis* and *An. coustani* complex feeding more slowly (< 35% fully engorged in approx. 40–60 min.)

Of the 5915 adult female mosquitoes allowed to feed on infected blood, 2106 (35.6%) fed successfully in the 32 independent parallel experimental infections. The feeding rate for *An. gambiae* s.l., *An. pharoensis* and *An. coustani* complex was 39.7% (1025 fed mosquitoes), 35.7% (594 fed mosquitoes) and 29.2% (487 fed mosquitoes), respectively. The proportion of fed mosquitoes after exposure to an infected blood meal varied significantly among species (G-test = 48.43, *df* = 2, *P* = 3.049e-11) (Table 1). The percentage of infected mosquitoes following feeding on an infected blood meal was significantly different among species (G-test = 6.4955, *df* = 2, *P* = 0.039) (Table 1).

**Table 1:** *Anopheles* species by their physiological status and infection rate

*Anopheles* mosquito species	Infecting* Plasmodium* species	Number of mosquitoes exposed	Number of fed mosquitoes (%)	Number of mosquitoes surviving and subsequently dissected (%)	Infection rate (%)
*An. gambiae* s.l	*P. vivax*	1775	723 (40.7%)	229 (31.7%)	188 (82%)
	*P. falciparum*	810	302 (37.3%)	108 (35.8%)	66 (61.1%)
	Total	2585	1025 (39.6%)	384 (37.5%)	254 (66.1%)
*An. pharoensis*	*P. vivax*	1170	435 (37.2%)	244 (56.1%)	157 (64.3%)
	*P. falciparum*	495	159 (32.1%)	111 (69.8%)	60 (54%)
	Total	1665	594 (35.7%)	355 (59.8%)	217 (61.1%)
*An. coustani* complex	*P. vivax*	1170	351 (30%)	225 (64.1%)	136 (60.4%)
	*P. falciparum*	495	136 (29.6%)	97 (71.3%)	47 (71.3%)
	Total	1665	487 (29.2%)	322(66.1%)	183 (56.8%)

*s.l.* Sensu lato

Overall, pairwise comparison showed that only significantly different IR was between *An. gambiae* s.l. (61.1%) and *An. coustani* complex (56.8%) (*P* < 0.01) (Table 1). For all other comparisons, the differences were non-significant.

### Measurement of II by the IDMFA

**Fig.1:**
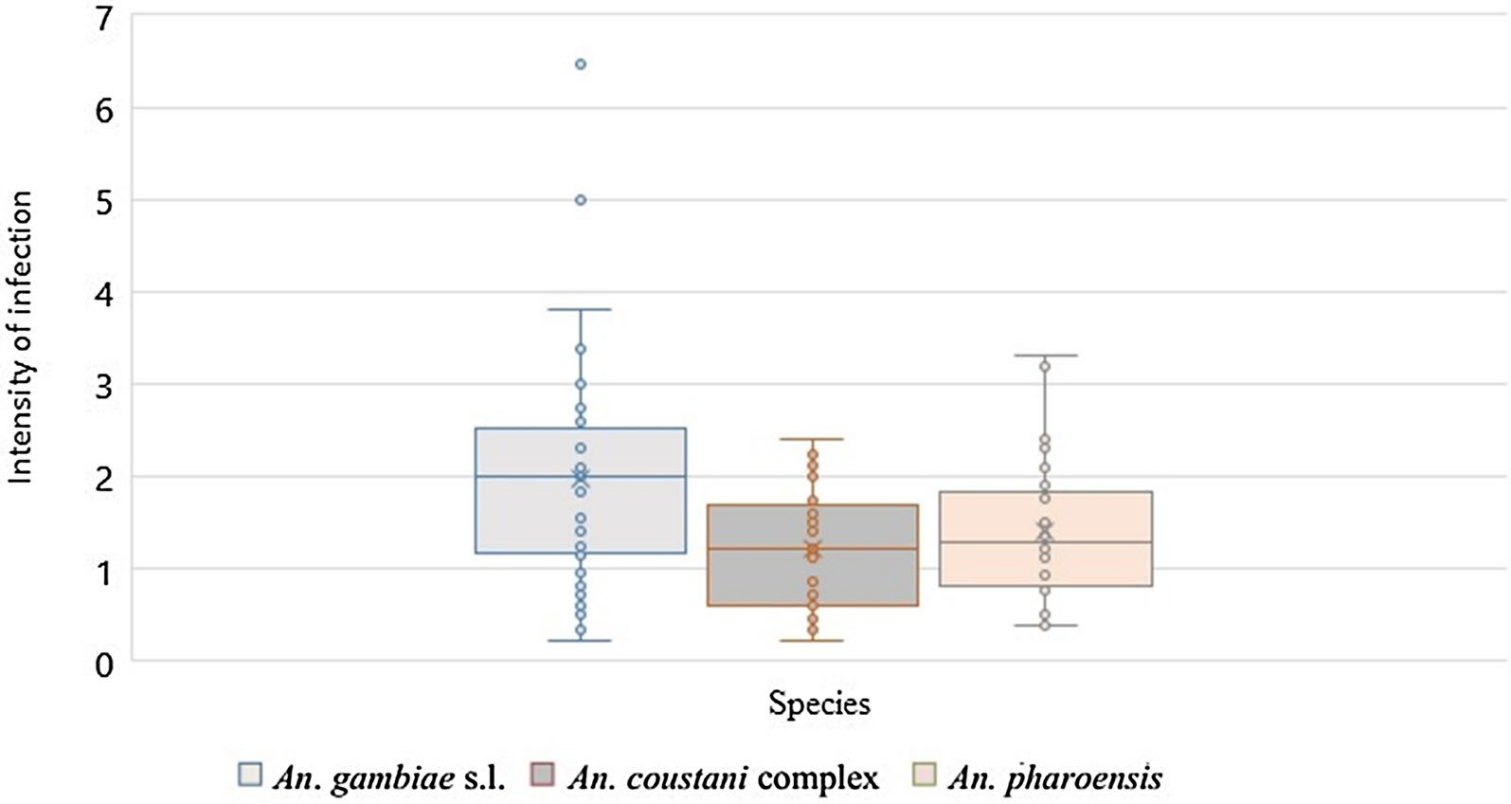
Box plot showing mean intensity of *Plasmodium *infection according to the indirect membrane feeding assay in populations of *Anopheles gambiae* sensu lato*, An. coustani* complex and *An. pharoensis*

The mean II was significantly different among *An. gambiae* s.l., *An. pharoensis* and *An. coustani* complex (*χ*^2^ = 10.709, *df* = 2, *P* < 0.005) (Fig. 1). However, pairwise comparisons using the Wilcoxon test indicated that the differences were only significant between *An. gambiae* s.l. and *An. coustani* complex (*P* < 0.005). The median II for the *An. coustani* complex was 1.16 compared to 2.00 for *An. gambiae* s.l*.* and 1.25 for *An. pharoensis*.

### Relationship between gametocyte abundance and oocyst density

**Fig. 2:**
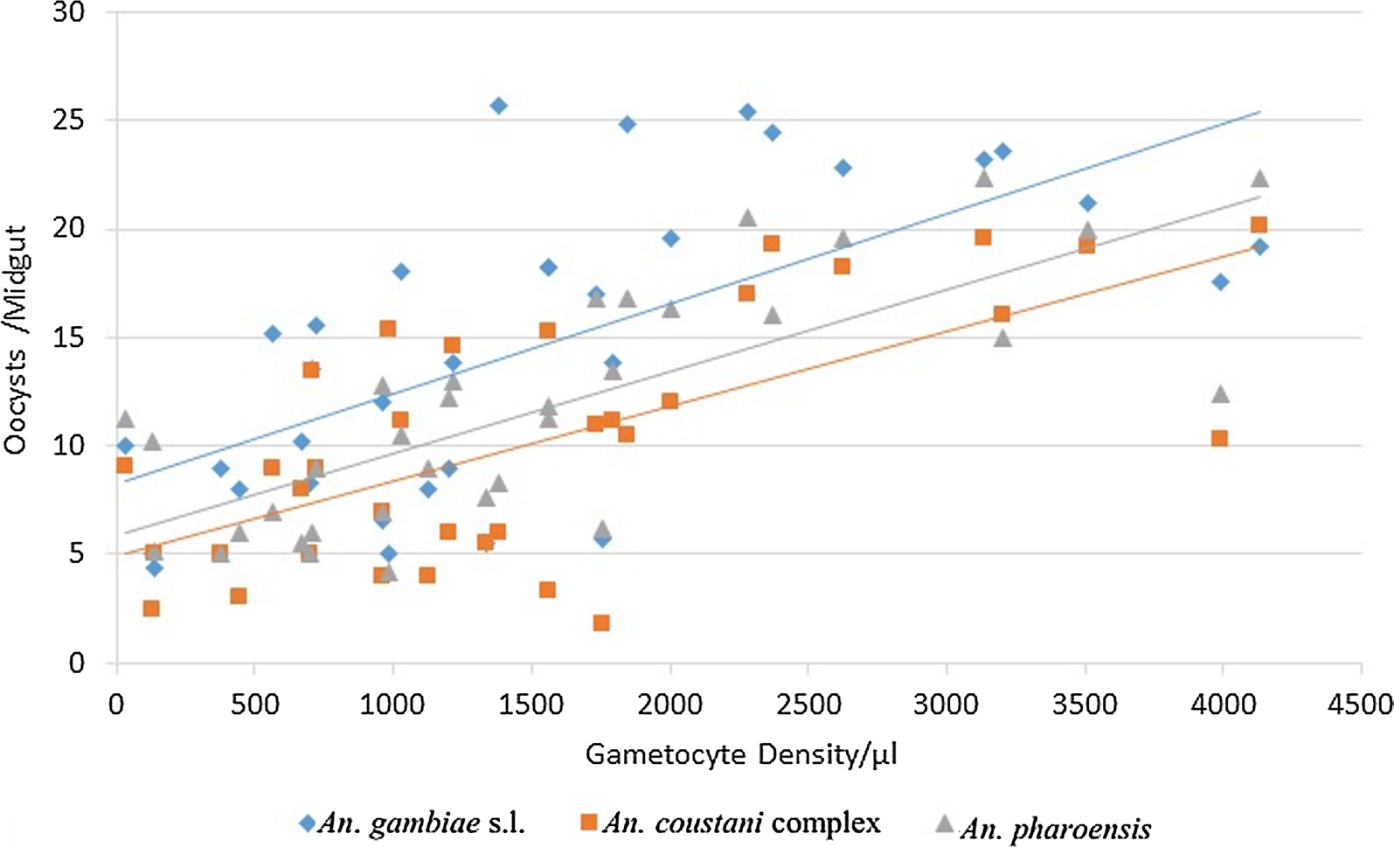
Correlation between mean oocyst and gametocyte abundance (density/μl) in populations of *An. gambiae* s.l*.*, *An. coustani* complex and *An. pharoensis*

There was a significant correlation between gametocyte and mean oocyst abundances (*P* < 0.001). Correlation comparison between gametocyte density and mean oocyst density showed a positive linear relationship although the association among species varied: *An*. *gambiae* s.l. (*r* = 0.65, *P* < 0.001), *An. pharoensis* (*r* = 0.66, *P* < 0.001) and *An. coustani* complex (*r* = 0.76, *P* < 0.001) (Fig. 2).

**Fig. 3:**
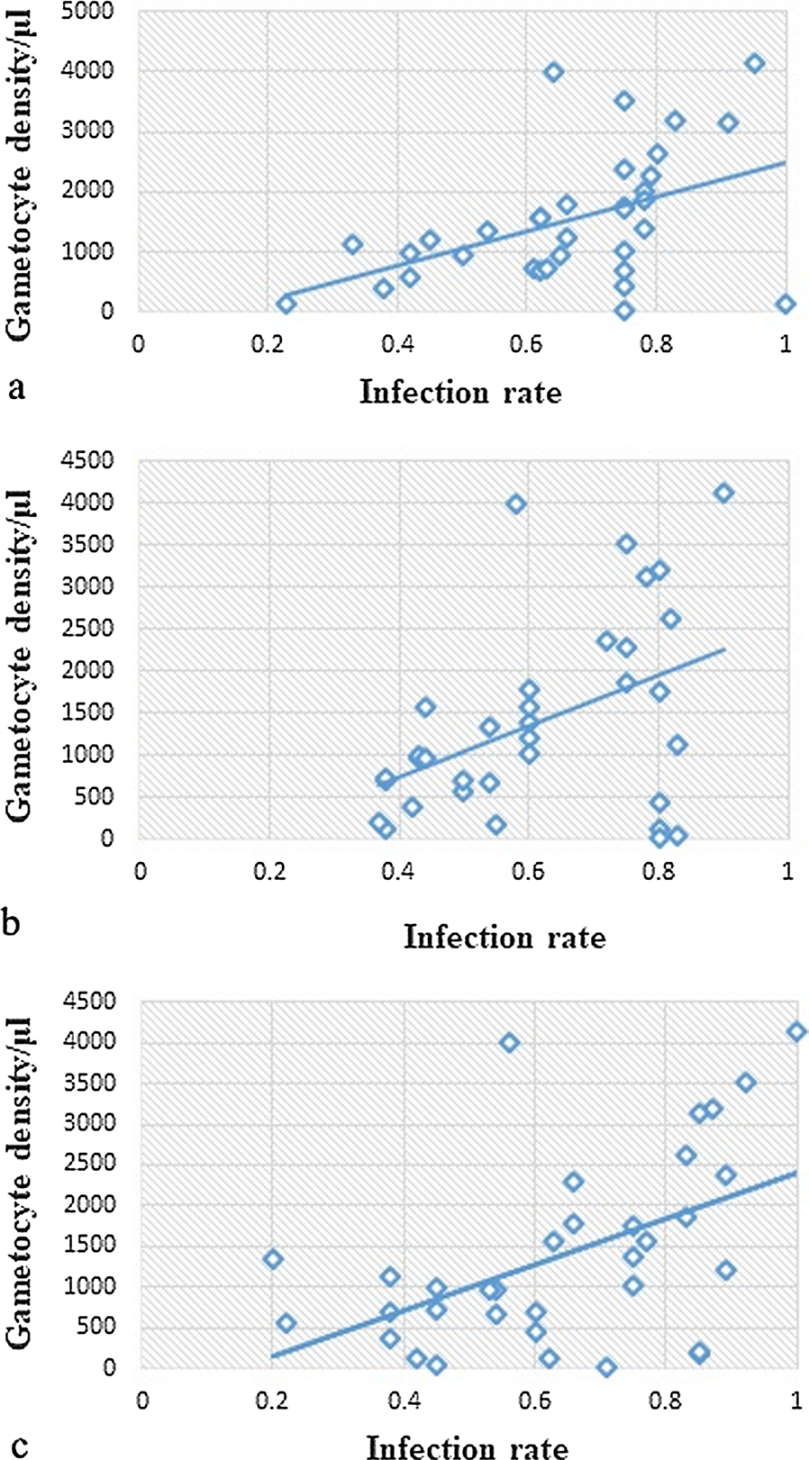
Correlation between infection rate and gametocyte density of *Plasmodium vivax* and *P. falciparum*. The results of the indirect membrane feeding assay, performed for individual feeds in parallel: **a** *An. gambiae* s.l., **b** *An. pharoensis*, **c** *An. coustani* complex

A positive correlation between the gametocytes/500 leukocytes and the IR of the mosquitoes was observed. However, the association varied among species: *An. gambiae* s.l. (*r* = 0.46, *P* < 0.01), *An. pharoensis* (*r* = 0.64, *P* < 0.001) and *An. coustani* complex (*r* = 0.44, *P* < 0.01) (Fig. 3).

## Discussion

Mosquitoes belonging to the *Anopheles coustani* complex showed susceptibility for the development of *P. falciparum* and *P. vivax* parasites in experimental infection assays. However, *An. gambiae* s.l. and *An. pharoensis* had a significantly higher IR than the *An. coustani* complex, which is suspected to be a malaria vector in Ethiopia. In addition, all three species showed high mean oocyst density, with the highest oocyst density found in *An. gambiae* s.l., which is one of the main malaria vector species in sub-Saharan Africa. The results of some studies in a number of countries conducted under natural conditions indicate that *An. coustani* complex supports the development of both *P. falciparum* and *P. vivax* [33, 34]. In contrast to our results, results from similar experimental studies conducted in Jimma town, southwest Ethiopia, indicated that *An. coustani* complex is not susceptible to *P. vivax* development [32]. This discrepancy might be due to differences in larval breading sites and the presence of different member species of *An. coustani* complex: i.e. different sibling species may be present in different parts of the country.

Defining the susceptibility of anopheline species to malaria parasites in time and space is vital for designing efficient vector control and elimination programs [31–33]. In this study, we documented a difference in susceptibility among the three species tested. The rate of mosquito infection is affected by various factors, such as environmental, biological and behavioral factors [19, 35, 36]. *Anopheles coustani* complex is a suspected vector in Ethiopia [33, 34]. Immunological studies using wild catch mosquitoes of *An. coustani* in different countries in sub-Sharan Africa have shown its susceptibility to *P. falciparum* and *P. vivax* infection [33–40].

The susceptibility of *An*. *gambiae* s.l. and *An. pharoensis* to infection by the malaria parasites *P*. *vivax* and *P. falciparum* has long been established [41]. A growing body of evidence, including this study, confirms the susceptibility of *An*. *coustani* complex to both *P. vivax* and *P. falciparum* from Ethiopia, Kenya, Zambia, Democratic Republic of Congo and Cameroon [18, 33, 36, 38, 42] where field populations were susceptible [33, 34, 43].

Vector competence varies from species to species, and there is usually a high proportion of uninfected mosquitoes when the infection process is controlled. This has been confirmed in studies conducted in Ethiopia and elsewhere [32, 44, 45]. On the other hand, the IR of the three species examined in the present study are much higher in experimental studies than under condition of natural infection. As indicated by studies employing enzyme-linked immunosorbent assays, based on the use of species-specific anti-sporozoite monoclonal antibodies, anopheline populations have varied IRs in sub-Saharan countries. For example, it has been reported that the IR of *An. gambiae* s.l ranges from 0.3% to 9.3%, that of An. *pharoensis* from 0.4% to 5.2% and that of *An. coustani* complex from 0.3% to 1.81% [18, 33, 46].

Gametocyte density has been found to be significantly correlated with IR. In our study, irrespective of the differences in species, we found that *Plasmodium* gametocyte density was significantly correlated to IR and mean oocyst density. This is also true in other studies conducted in Manaus, in the western Brazilian Amazon and Bengbu, Anhui Province, central China [44, 45].

Infected blood with equivalent gametocyte density was provided to the three species of mosquitoes, but variable susceptibility was observed. This discrepancy in IR might be due to different factors, such as gametocyte maturity, gametocyte sex ratio, different *Plasmodium* genotypes, rhythms in the density and infectivity of transmission forms (gametocytes), immune factors in patient sera and/or mosquitoes’ innate immunity. All of these factors could alter gametocyte infectivity [47–50]. In addition, recent studies indicate that sub-microscopic gametocyte density is capable of infecting mosquitoes, which demonstrates that rather than gametocyte density, the above contributing factors play a role in the variability of the IR and mean oocyst density. Studies from different countries show that sub-microscopic infections might be the major contributor to malaria transmission [51–54], suggesting that gametocyte density is not the only factor that determines infection in mosquitoes.

Furthermore, secondary, and suspected vectors, such as *An. pharoensis* and *An. coustani* complex, have a relatively higher abundance during the dry season [12]. This relatively higher abundance in the dry season may be an important factor in terms of local malaria transmission it may help to increase or prolong the malaria transmission period [55]. Secondary vectors are most often outdoor biting and outdoor resting mosquitoes [46, 56]. The role of outdoor-resting* Anopheles* in malaria transmission is important as these secondary vectors contribute to malaria transmission in Africa, and their role in transmission is not negligible [56, 57]. Most secondary vectors have a short survival rate, with 50–60% natural mortality rates per gonotrophic cycle [23, 24]. This might be the reason that the population density of the secondary vector has been low in many settings.

Most *Anopheles* vectors found in nature have only a few oocysts, and oocysts are of little importance in malaria epidemiology. Rather, it is the outdoor biting and resting behavior of mosquitoes in nature that contribute to residual malaria transmission in different parts of sub-Saharan Africa and which pose new challenges as they cannot be consistently controlled using current tools. The proportion of outdoor biting mosquitoes has been reported to have increased by 10% [58–60]. More recent studies in sub-Saharan Africa have indicated that *An. pharoensis* and *An. coustani* complex have likely become primary role in malaria transmission, possibly because existing interventions target primary vectors only to achieve complete malaria control [61].

## Conclusions

In the present study, we confirmed that primary, secondary and suspected vectors are receptive for the development of *P. falciparum* and *P. vivax* in this experimental infection model using IDMFA. For effective malaria elimination, there should be a focus on secondary and suspected vectors to avoid focal malaria transmission or limit residual malaria transmission. In addition, it is highly recommended that current vector surveillance and control tools be re-considered to target secondary and suspected vectors that may have an outdoor transmission role. In addition, further investigation of the ecology and dynamics of immature stages can improve our understanding of the bionomics of the receptiveness of these mosquitoes to *Plasmodium* development.

## Data Availability

The datasets used in this study are available upon reasonable request from the corresponding author.

## Abbreviations

ICEMR: International Center Excellence for Malaria Research
IDMFA: Indirect membrane feeding assay
II: Infection intensity
IR: Infection rate
